# Speckle Tracking Analysis Reveals Altered Left Atrial and Ventricular Myocardial Deformation in Patients with End-Stage Liver Disease

**DOI:** 10.3390/jcm10050897

**Published:** 2021-02-24

**Authors:** Franzisca von Köckritz, Alexander Braun, Rosa B. Schmuck, Eva M. Dobrindt, Dennis Eurich, Frank R. Heinzel, Burkert Pieske, Felicitas Escher, Kun Zhang

**Affiliations:** 1 Department of Internal Medicine and Cardiology, Campus Virchow-Klinikum, Charité—Universitätsmedizin Berlin, Augustenburger Platz 1, 13353 Berlin, Germany; franzisca.koeckritz@charite.de (F.v.K.); alexander.braun2@charite.de (A.B.); frank.heinzel@charite.de (F.R.H.); burkert.pieske@charite.de (B.P.); felicitas.escher@charite.de (F.E.); 2 Department of Surgery, Campus Charité Mitte and Campus Virchow Klinikum, Charité—Universitätsmedizin Berlin, Augustenburger Platz 1, 13353 Berlin, Germany; Rosa.schmuck@charite.de (R.B.S.); eva.dobrindt@charite.de (E.M.D.); dennis.eurich@charite.de (D.E.); 3 DZHK (German Centre for Cardiovascular Research), Partner Site Berlin, 10785 Berlin, Germany; 4 Berlin Institute of Health (BIH), 10178 Berlin, Germany; 5 German Heart Center Berlin, Department of Internal Medicine and Cardiology, 13353 Berlin, Germany

**Keywords:** cirrhotic cardiomyopathy, left ventricle, left atrium, speckle tracking, strain

## Abstract

Background: Cardiac function can be influenced by liver cirrhosis and should be thoroughly evaluated before liver transplantation. We investigated left ventricular (LV) and, for the first time, left atrial (LA) strain and strain rate in end-stage liver cirrhosis patients of different etiologies. Methods: This retrospective, cross-sectional study evaluated left heart function in 80 cirrhosis patients and 30 controls using standardized echocardiographic techniques and speckle tracking technology (STE) analysis. Serum markers of liver function were used for correlation analysis. Results: While conventional parameters demonstrated no alteration in systolic function, speckle tracking analysis showed a significant increase in LV longitudinal strain throughout all cardiac layers, with significant correlation to model of end-stage liver disease (MELD) score. LA reservoir and conduit strain as well as LA strain rate in all phases were significantly reduced in end-stage liver disease (ESLD) patients compared to control. STE for the evaluation of LA phasic function seemed to be more sensitive than volumetric methods. Kaplan-Meier curves showed a trend towards reduced post-transplant survival in patients with a reduced LA reservoir and conduit strain. Conclusion: STE analysis detected increased LV and decreased LA deformation in cirrhosis patients, thus proving to be highly sensitive to cardiac changes and useful for more precise cardiac evaluation.

## 1. Introduction

### 1.1. Cirrhotic Cardiomyopathy

Cirrhotic cardiomyopathy (CCM) is cardiac dysfunction in patients with end-stage liver disease in the absence of prior heart disease [1,2,3]. Mild CCM usually remains asymptomatic at rest and most commonly manifests as prolonged QT interval ECG abnormality and sole diastolic dysfunction seen in 2D imaging [2,3,4]. Diagnosis of CCM is frequently missed or delayed due to its asymptomatic state and seemingly normal cardiac function at rest [2,4,5,6].

However, acute stress conditions, such as infection, transjugular intrahepatic portosystemic shunt (TIPS) procedure, or liver transplantation (LTx), can lead to extreme forms of CCM with overt heart failure in patients with end-stage liver disease (ESLD) [4,5]. Transthoracic echocardiography (TTE) is required by the European Association for the Study of the Liver to unmask previously unknown cardiological conditions [5]. More elaborate evaluations (i.e., using dobutamine stress echocardiography) are usually only performed if abnormal TTE results were collected or if the patient’s profile presents certain risk factors [4,5]. Estimates of ESLD patients who underwent LTx with an unknown cardiovascular disease are as high as 25–50% [2,3,4,5,6], and cardiovascular events account for approximately 7–21% of the subsequent deaths [5,7]. Thus, other more sensitive diagnostic approaches in cardiac imaging should be considered when evaluating cardiac functions of ESLD patients [5]. 

### 1.2. Speckle Tracking Echocardiography

Two-dimensional speckle tracking echocardiography (2D-STE) is a new technique to assess cardiac function, with major focus on the left ventricle (LV) in the clinical setting. It uses grayscale digital images with speckle patterns obtained from sonographic procedures such as TTE and analyzes the relative displacement of individual speckles on a frame-to-frame basis to quantify myocardial deformation, also referred to as “strain” [3,5,8,9]. Strain rate (SR) is the thickening or shortening per time and is yet another tool to assess myocardial function [3,5]. In the updated criteria for diagnosis of cirrhotic cardiomyopathy by the Cirrhotic Cardiomyopathy Consortium (CCC), the evaluation of LV global longitudinal strain (GLS) in addition to left ventricular ejection fraction (LVEF) has been proposed in order to estimate systolic function [1,10]. 

To date, STE has only been validated for LV assessment [11]. However, in recent years, the established software has also been used for left atrial (LA) evaluation as it allows detailed judgment of LA phasic reservoir, conduit, and contractile functions [11,12]. In comparison to volumetric measurements used conventionally for phasic assessment, LA strain has demonstrated higher sensitivity in early stages of disease, specifically in the assessment of diastolic function [9,12,13,14].

### 1.3. Aim 

The present retrospective study aims to evaluate left ventricular and atrial myocardial deformation in patients waiting for liver transplantation using speckle tracking technology. The novel aspect of this study is the detailed evaluation of left atrial strain and strain rate in patients with end-stage liver disease.

## 2. Methodology

### 2.1. Patients and Control Group

From an archive of 290 LTxs performed in the Charité Campus Virchow center from 2013 until 2016, 80 ELSD patients with normal LVEF were considered for echocardiographic speckle-tracking analysis. Patients with a history of coronary disease, heart failure, congenital heart disease, atrial fibrillation, or moderate-to-severe valvular disease were excluded from the study. Thirty patients matched for age and sex, without liver disease, served as the control group.

Anthropometric measures (height and weight) were recorded for subjects and used to calculate body mass index (BMI) and body surface area (BSA). Laboratory analysis, including liver enzymes aspartate aminotransferase (ASAT), alanine aminotransferase (ALAT), alkaline phosphatase (AP), and gamma-glutamyltransferase (γGT); prothrombin time as the international normalized ratio (INR); total serum bilirubin; and creatinine, was performed on blood samples from all subjects on the day of echocardiography ± 10 days, except for one patient, whose lab results were taken 33 days before. We calculated the model for end-stage liver disease (MELD) score according to conventional formula using creatinine, bilirubin, and the INR. Values less than 1.0 were set to 1.0 for the purposes of calculation. The study was approved by the local ethics committee (#EA4/065/19). 

### 2.2. Echocardiography and STE

TTE examinations were performed in the abovementioned timeframe by specialized cardiologists using Vivid 7 Ultrasound (GE Vingmed, Horton, Norway). Echopac 201 software (GE-Healthcare, Horton, Norway) was used to store TTE images and provided necessary tools for analysis and 2D STE. Standard echocardiographic images were recorded in parasternal short and long axes and apical two, three, and four chamber views using 2D echography. These were used to evaluate LV and LA dimensions and function utilizing established and endorsed techniques (i.e., caliber and volumetric measurements). The collected values permitted further calculations of relative wall thickness (RWT), LV mass (LVM) and mass index (LVMI), and LA volume index (LAVI). Additionally, LA phasic emptying volumes were calculated for the reservoir (TotEV), conduit (PassEV), and contractile (ActEV) phases. All values were then indexed (TotEF, PassEF, and ActEF) according to Andrew et al. [15]. LV ejection fraction (LVEF) data were obtained from statements recorded by examiners. 

Furthermore, a pulse-waved (PW) doppler was used during TTE exams, allowing assessment of LV diastolic function through quantification of transmitral inflow velocities during early (E) and late (A) diastole and deceleration time (DT) [16]. The E/A ratio was computed. Septal and lateral mitral annular diastolic velocities (e’ septal/lateral) were also collected using the PW doppler. Average e’, derived from septal and lateral e´ values, was used to calculate the E/e´ ratio [16]. 

STE was performed using three consecutive cycles. Total region of interest (ROI) was manually traced for the software to recognize individual regions automatically. If the system did not identify all regions or tracking was visually inadequate, manual adjustments were made to ROI [9]. We accepted a maximum of one region not being identified by the system. LV GLS strain was computed in apical two-, three, and four-chamber (2CH, 3CH, 4CH) views. For LV global circumferential strain (GCS) and global radial strain (GRS) analysis, images in the parasternal short axis at the level of the papillary muscle were used. LA was analyzed in 2CH and 4CH views. LA strain and SR used systolic gating processing, beginning STE measurements with the onset of the QRS complex [11]. LA strain was given graphically by Echopac software. This allowed measuring peak atrial longitudinal strain and peak atrial contraction strain for the reservoir and contractile phasic function accordingly [11,12]. The calculated difference between the two was interpreted as atrial conduit function [11,12]. LA SR was also given graphically, and values for all the previously mentioned phases (SRs—reservoir, SRe—conduit, and SRa—contractile function) were obtained by measuring peak SR at systole and early and late diastole [11]. 

### 2.3. Statistical Analysis

Statistical analysis was performed with IBM SPSS Version 25 (SPSS Inc., Chicago, IL, USA) for Windows. All variables were checked for normal distribution graphically and using the Kolmogorov-Smirnov test. Consequently, the Mann-Whitney U or *t*-test were applied when appropriate. The Kruskal-Wallis test was used to compare GLS for different etiologies of cirrhosis. Spearman’s correlation coefficient (*r*) was used to analyze any existing relationship between clinical, STE, and LA phasic function parameters in patients. Survival functions (Kaplan-Meier estimator) were computed to show differences in mortality for ESLD patients according to LA strain. Finally, we assessed predictors for mortality using Cox regression analysis.

Results are shown as average mean ± standard deviation and are accepted as statistically significant when *p* < 0.05. 

## 3. Results

### 3.1. Demographic and Clinical Data

Eighty ESLD patients (47 males and 33 females) and a control group of *n* = 30 (14 males and 16 females) were recruited for this study. Controls had undergone TTE procedures as evaluation for potential organ or tissue donations or as regular checkup. 

The basic demographic and clinical parameters are summarized in Table 1. As expected, liver enzymes ALAT, ASAT, and γGT and laboratory markers creatinine, total bilirubin, and INR were all significantly elevated in patients. Hence, the calculated average MELD score was significantly increased (*p* < 0.001). 

Patients suffered cirrhosis due to various etiologies. These included alcoholism (31.25%), hepatitis C (12.5%), autoimmune (10%), Non-Alcoholic Steatohepatitis (NASH 10%), Primary Sclerosing Cholangitis (PSC, 8.75%), idiopathic (8.75%), and others (e.g., cystic liver, M. Wilson, bile duct carcinoma, and Caroli syndrome; 18.75%). Due to the retrospective nature of this study, severity of disease was not classified using the Child-Pugh score. 

At the time of this retrospective study, all patients had undergone LTx at our transplantation center. Up until finalizing data collection (Nov. 2020) 65 patients were still alive, 13 had died, and the status of 2 patients was unknown, as no recent update nor death records were registered. Of the deceased, an average time of survival of 20 months was calculated.

### 3.2. Echocardiography and Strain Measurements of the Left Ventricle

ESLD patients presented increased left ventricular mass (LVM) and end-diastolic dimension (LVEDD), as well as interventricular septum (IVS) and posterior wall thickness (PW) in comparison to controls. However, no statistically relevant changes were observed in width of aortic sinus, LV end-systolic dimension (LVESD), or RWT. Most importantly, both groups were found within a normal range concerning LVEF, with healthy controls and ESLD patients averaging at 60.90 ± 4.70% and 60.00 ± 5.17% (*p* = 0.274), respectively. End-diastolic and end-systolic volumes (EDV and ESV) were also unaltered.

Mitral inflow velocities (E, A), deceleration time (DT), and peak mitral annular velocity parameters (E´ septal, E´ lateral) were collected for patients and controls when appropriate echocardiographic images were available for evaluation. DT and the calculated E/A ratio, relevant for diagnosis of diastolic dysfunction, showed no difference between the two groups and were within normal ranges (DT < 140 ms and E/A > 0.8) according to ASE/EACVI Guidelines and Standards [16]. E´ values for septal and lateral mitral annulus points were also collected and did not differ among populations. The E/e´ ratio was calculated using an average of septal and lateral measurements and was significantly higher for ESLD patients at 9.42 (*p* < 0.005). 

**Figure 1:**
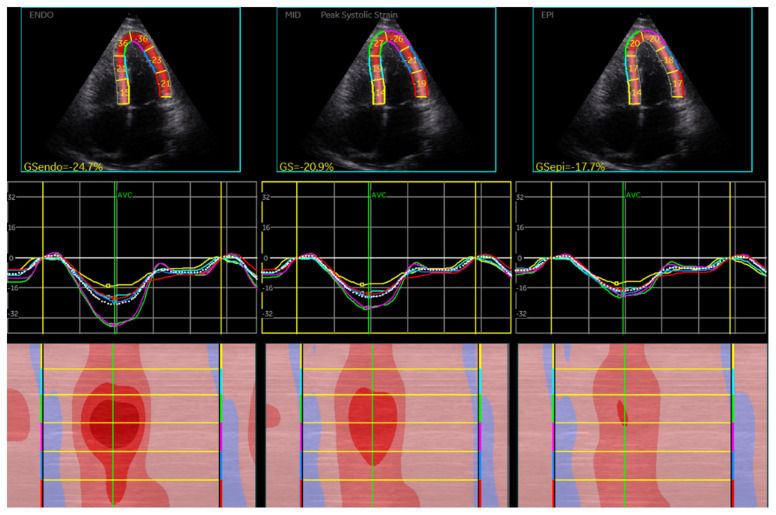
Example of left ventricular (LV) multilayer global longitudinal strain (GLS) in transthoracic echocardiography (TTE) four-chamber view.

As seen in Table 2, patients displayed overall a significantly higher average GLS (*p* < 0.001). Mid-myocardial and endo- and epi-cardial layers similarly showed significantly higher values, suggesting increased transmural movement, as illustrated in Figure 1. GCS and GRS results showed no significant changes. Standard deviation was higher in GRS than in other strain analyses. 

GLS analysis subdivided into different cirrhosis etiologies showed no significant difference between the groups (Table 3). Of note, subgroups partly contain a limited number of patients.

### 3.3. Echocardiography and Strain Measurements of the Left Atrium

Volumetric echocardiographic results are not reported for each patient and control due to ECG quality (i.e., p-wave not precisely distinguishable) or missing BMI in one patient, thus limiting the available data points. Nonetheless, elevated LA volumes were recorded at the end of ventricular systole (i.e., maximum dilation), beginning of p-wave, and end of ventricular diastole (i.e., minimal contraction) in ESLD patients, reaching statistical relevance with *p* < 0.001 for all three values. Consequently, all calculated values for LAVI reached similar significance when comparing patients to controls. 

All LA phasic volumetric values showed a consistent significant increase for patients. However, this change was not seen when values were indexed, as only the LA passive emptying fraction (i.e., conduit phase) reached statistical significance, while LA total and active emptying fractions (i.e., reservoir and contractile phases) showed no difference between the two groups (Table 4). 

**Figure 2:**
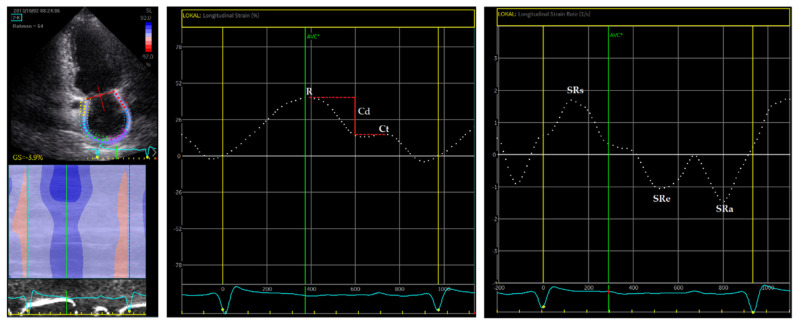
Example of LA strain and strain rate measurements measured in TTE two-chamber view. R—reservoir strain, Ct—contractile strain, Cd—conduit strain, SRs—reservoir strain rate, SRe—conduit strain rate, SRa—contractile strain rate.

Speckle tracking analysis was preformed likewise for all three atrial phases and demonstrated reduced LA strain in reservoir and conduit phasic functions with *p* = 0.002 and *p* < 0.001, respectively. LA contractile strain was unaltered. A significant decrease in strain rate was observed in ESLD patients for all atrial phases. Speckle tracking analysis is exemplarily depicted in Figure 2.

### 3.4. Prevalence of CCM in Study Cohort

When applying the criteria proposed by the CCC [1], 27.5% of the patients could be diagnosed with CCM. Among the patients, 14/80 (17.5%) showed systolic dysfunction, yet rather mild (average LVEF: 57%; average GLS: 16%). Regarding diastolic function, nine patients presented indeterminate function after initial evaluation. Further evaluation of these patients using LA strain according to the CCC recommendations was performed. Ultimately, 58 patients presented normal diastolic function, 12 grade I (15%), 7 grade II (8.75%), and 3 grade III (3.75%) dysfunction. Interestingly, 62% of the patients with normal diastolic function also showed a reduced LA reservoir strain. 

### 3.5. Correlation Analysis in ESLD Patients 

Correlation analyses are summarized in Table 5. The most noteworthy correlations found were concerning GLS. All LV global longitudinal strains measured (epi-, endo- and mid-myocardial) consistently correlated with ALAT, ASAT, bilirubin, and the INR, each to an extent of *p* < 0.01. This indicates that higher laboratory markers are associated with increased GLS (more negative) values. Hence, correlations between the endo-, epi- and mid-myocardial GLS and MELD are comprehensible since MELD score is based upon previously mentioned laboratory markers. GLS of individual myocardial layers correlated with MELD to a similar extend, with epicardial reaching *r* = −0.379, mid-myocardial *r* = −0.360, and endocardial *r* = −0.346.

In addition, correlation analysis of LA parameters with laboratory markers and MELD were computed. The results show that LA strain and strain rate tend to correlate comparatively consistently with liver enzymes ALAT and ASAT, creatinine and the INR when compared with LA phasic volumetric parameters.

### 3.6. Mortality Analysis in ESLD Patients

**Figure 3:**
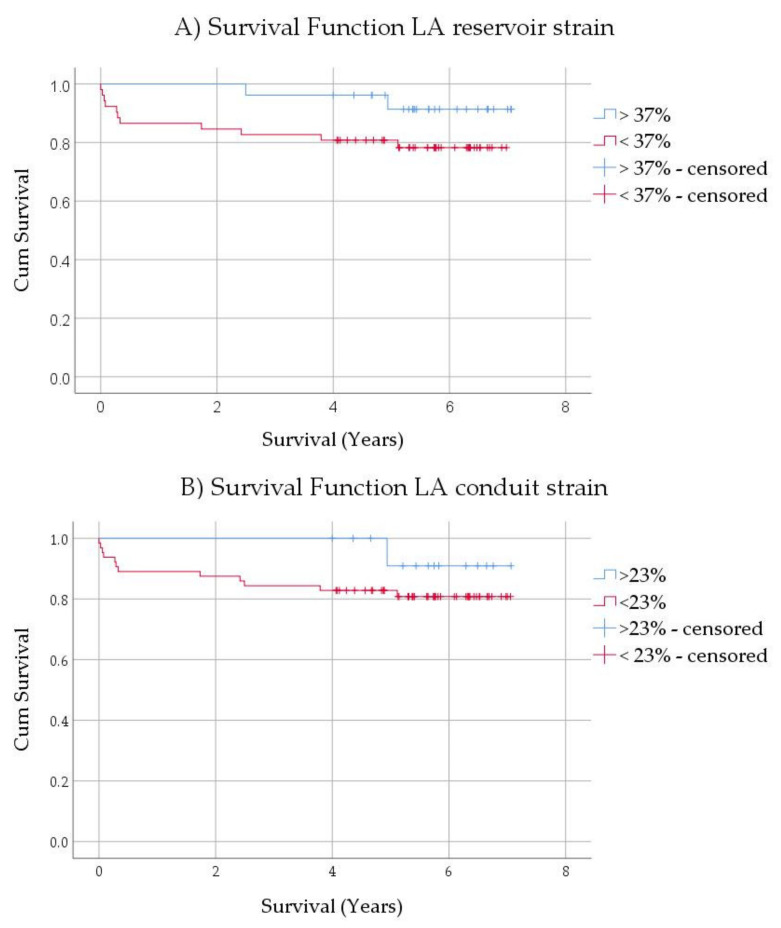
Kaplan-Meier curves for left atrial (**A**) reservoir (*p* = 0.123) and (**B**) conduit (*p* = 0.286) strain, showing trend towards increased post-transplant mortality. LA: left atrium

Kaplan-Meier curves were computed for LA reservoir, conduit, and contractile strain. While none reached statistical significance, it should be noted that reservoir (*p* = 0.123) and conduit (*p* = 0.286) strain demonstrated a trend towards patients with pathological values being at higher risk of mortality after LTx (Figure 3). Perhaps the sample size remains too small. No such trend was observed for contractile strain (*p* = 0.434). Similarly, Cox regression analysis identified none of the tested variables as predictors of mortality: LA reservoir (*p* = 0.099), conduit strain (*p* = 0.236), contractile strain (*p* = 0.432), GLS (*p* = 0.993), MELD (*p* = 0.797), and BMI (*p* = 0.921).

## 4. Discussion

The present study evaluated left heart function in ESLD patients using established echocardiographic techniques and extending this inquiry by employing 2D speckle tracking technology. This study presents the first evaluation of left atrial strain and strain rate in ESLD patients.

Our results indicate significantly elevated GLS for ESLD patients, while LVEF values were unaltered. Data on GLS in cirrhotic patients with normal LVEF are limited and conflicting [17,18,19,20]. Mechelinck et al. recently reported that both low and high GLSs occur in ELSD patients and are both negative prognostic factors [20]. While a reduced GLS represented a subclinical systolic dysfunction, an increased GLS was associated with more advanced liver diseases [20]. Supporting this finding, we established a correlation of LV GLS with various liver-specific laboratory markers and MELD score. Kim et al. observed normalization of elevated GLS in cirrhosis within one-year post-transplantation, showing the therapeutic value of LTx for systolic function [19].

The deformation of cardiac fibers throughout all layers illustrates the increased myocardial stress in the context of a hyperdynamic circulatory syndrome, which has long been described in cirrhotic patients and is attributed to the heart’s response to splanchnic arterial vasodilation and decreased systemic vascular resistance [2,8,21,22]. Transmural, systolic activation detected in this study can also be additionally supported by elevated GCS and GRS trends when comparing patients to controls, although results do not reach statistical relevance. In addition to ventricular deformation changes, we observed an increase in LVEDD, IVS, PW, and LVM, which suggests cardiac remodeling in ESLD patients [23,24,25]. Patients presented with significantly increased LVMI (102.49 ± 28.50 vs. 79.87 ± 21.99 g/m^2^), thus trending towards concentric hypertrophy [26].

Based on the accumulating evidence that GLS is a useful value for the assessment of systolic function, the recently updated criteria for the diagnosis of CCM by the CCC included the evaluation of GLS (normal range absolute GLS ≥ 18%) in addition to LVEF (normal range >50%) [1,10].

DD has been described as an early marker for CCM [8] and can be identified through four conventional variables: mitral annular velocities (e´ septal and lateral), E/e´ratio, LAVI, and tricuspid regurgitation velocity (TR vel) [16]. According to the ASE/EACVI guidelines and as it has also been proposed by the CCC, diastolic function, as well as its severity, is diagnosed based on these four criteria [1,16]. Considering our results of the parameters, only LAVI was significantly elevated. With an E/A ratio >0.8, normal diastolic function can be stated according to the abovementioned guidelines. The E/e´ ratio also showed a significant increase; however, with a value of 9.42 ± 2.88, patients presented indeterminate results which do not allow clear interpretation [16,19,27]. Hence, diagnosis of DD in ESLD patients could not be validated through conventional parameters.

STE analysis of the left atrium has been proposed as an alternative approach for LV filling pressure and diastolic function assessment, as it displays the physiology of left atrial function which closely follows LV dynamics [28,29]. Remodeling of the LA has been proposed as a measure of diastolic burden and a predictor of cardiovascular outcomes such as new atrial fibrillation, heart failure, or cardiovascular death [29]. Our study demonstrated significant changes in left atrial strain and strain rates (SR) in cirrhosis. While volumetric measurements could not discriminate a convincing difference in LA phasic function, LA reservoir and conduit strain as well as strain rate in all phases were significantly reduced in ESLD patients compared to control. In agreement with this finding, several studies reported that myocardial LA analyses using STE have advantages over volumetric LA measurements [30,31].

In the current recommendations for the diagnosis of CCM by the CCC, the evaluation of LA strain was included to further evaluate patients with indeterminate diastolic function [1]. In this study, nine patients presented indeterminate function after initial evaluation, out of which four were categorized in advanced diastolic dysfunction (grade II or III) with the use of LA strain. However, a substantial number of patients with normal diastolic function (62%) also showed a reduced LA reservoir strain. Reduced LA strain has been associated with heart failure with preserved EF (HFpEF) and is suggested as an early marker of such, as conduit and reservoir functions decline prior to definite diagnosis of DD [32], thus suggesting increased sensitivity compared to conventional parameters [11,12,31,32]. This is supported by our results as both reservoir and conduit strain are reduced significantly, while standard echocardiographic measurements are too uncertain to diagnose DD. Moreover, Kaplan-Meier curves showed a trend towards reduced post-transplant survival in patients with reduced LA reservoir and conduit strain. Considering the results of this study and the previous reports in the literature, it can be discussed whether LA strain should belong to the parameters for the initial evaluation of diastolic function. However, data on LA strain and strain rate in cirrhotic patients are very scarce. In concordance with our results, Sampaio et al. reported a reduced LA reservoir strain and an unchanged LA contractile strain in cirrhotic patients [33]. To the best of our knowledge, this is the first time that LA strain rate analysis was conducted in cirrhotic patients. Like LA strain, a decrease in LA strain rate is associated with increased LV filling pressure and different conditions of heart disease, according to Gan et al. [11]. There are as of yet no validated algorithms and established normal values.

This study demonstrates that STE analysis of LA deformation exposes minor cardiac dysfunction in ESLD patients more accurately than conventional measurements and should be further evaluated during cardiac evaluation. To gain more insight, more studies on this subject including the evaluation of outcome parameters are needed.

## 5. Limitations

The most prominent limitation of this study is its retrospective nature. This limited the amount of obtained laboratory data and cardiac imaging.

In consideration of previously reported potential changes in GLS post LTx by Kim et al. [19], the lack of follow up cardiac examinations is a great limitation. These were not available for patients since they were transferred into ambulant care for annual checkups. Furthermore, the study at hand was a single-center study, which allowed for only a small number of patients and controls to be enrolled. Even though significant differences in cardiac function were observed, further investigations with a larger study cohort would be necessary to confirm the obtained results. Additionally, considering our small population, we did not focus on grouping patients according to etiology of cirrhosis. Lastly, although 2D-STE allows for angle-independent myocardial deformation and shows higher sensitivity than conventional parameters, it still presents high inter-vendor variability [9], thus requiring reference values established with the same system.

## 6. Conclusions

In brief, this study demonstrates that strain analysis of the left ventricle and atrium is a useful tool to detect subtle changes in left ventricular systolic and diastolic function in patients with end-stage liver disease. Using 2D-STE, we were able to demonstrate increased LV GLS and an impairment of LA atrial strain and strain rate in cirrhotic patients.

## Figures and Tables

**Table 1:** Demographic and clinical data of healthy controls vs. end-stage liver disease (ESLD) patients.

Variables	*n*	Controls			*n*	ESLD Patients			Statistics *p*
Gender (Male %)	30	46.70			80	58.80			0.258
Age (years)	30	48.57	±	12.93	80	52.47	±	10.24	0.145
BMI (kg/m^2^)	30	24.43	±	3.40	79	26.30	±	5.11	0.067
ALAT (U/L)	30	23.63	±	10.19	80	58.39	±	48.49	<0.001
ASAT (U/L)	30	27.73	±	21.51	80	81.48	±	73.86	<0.001
AP (U/L)	29	66.34	±	18.01	80	235.91	±	376.25	<0.001
γGT (U/L)	30	21.40	±	11.80	80	159.75	±	212.32	<0.001
Creatinine (mg/dL)	30	1.02	±	0.06	80	1.16	±	0.33	0.028
Bilirubin (mg/dL)	29	1.02	±	0.06	80	7.01	±	8.64	<0.001
INR	29	1.02	±	0.04	80	1.55	±	0.52	<0.001
MELD Score	29	7	±	0.70	80	17	±	6.65	<0.001

BMI—body mass index, ALAT—alanine aminotransferase, ASAT—aspartate aminotransferase, AP—alkaline phosphatase, γGT—gamma-glutamyltransferase, INR—international normalized ratio, MELD—model for end-stage liver disease.

**Table 2:** Echocardiographic parameters of the left ventricle.

Variables	*n*	Controls			*n*		ESLD Patients			Statistics *p*
Aorta sinus (mm)	30	29.53	±	3.60	78	30.56		±	3.49	0.176
LVEDD (mm)	30	44.80	±	5.74	80	47.59		±	6.63	0.045
LVESD (mm)	30	34.20	±	4.47	80	35.75		±	9.22	0.416
IVS (mm)	30	9.50	±	1.59	80	11.12		±	1.84	<0.001
PW (mm)	30	9.77	±	1.52	80	11.05		±	1.89	0.001
RWT	30	0.44	±	0.07	80	0.47		±	0.10	0.121
LVM (g)	30	149.27	±	51.96	80	199.11		±	62.34	<0.001
LVMI (g/m^2^)	30	79.87	±	21.99	79	102.49		±	28.50	<0.001
LV Volume_max_	30	107.43	±	31.03	80	118.50		±	38.52	0.161
LV Volume_min_	30	59.20	±	22.73	80	57.11		±	20.24	0.642
E (m/sec)	29	0.71	±	0.16	75	0.80		±	0.22	0.058
A (m/sec)	29	0.59	±	0.17	75	0.70		±	0.23	0.035
E/A ratio	29	1.25	±	0.36	75	1.23		±	0.41	0.828
DT (ms)	29	214.10	±	50.59	75	236.43		±	56.92	0.068
e’ sept (m/sec)	28	0.13	±	0.20	75	0.08		±	0.02	0.188
e’ lat (m/sec)	28	0.10	±	0.03	69	0.10		±	0.02	0.984
E/e’ ratio	28	7.57	±	2.88	69	9.42		±	2.88	0.005
TR (m/sec)	16	2.41	±	0.33	65	2.53		±	0.38	0.232
LVEF (%)	30	60.90	±	4.70	79	60.00		±	5.17	0.274
**Strain Analysis (%)**										
GLS average	30	−18.73	±	2.95	80	−21.39		±	4.06	<0.001
GLS mid-myocardial	30	−18.56	±	2.63	80	−21.26		±	4.05	<0.001
GLS endocardial	30	−21.34	±	3.00	80	−24.16		±	4.58	<0.001
GLS epicardial	30	−16.28	±	2.39	80	−18.75		±	3.62	<0.001
GCS	22	−17.16	±	5.06	55	−19.85		±	6.69	0.093
GRS	22	30.77	±	21.22	55	35.92		±	18.79	0.298

LV—left ventricle, LVEDD—end-diastolic diameter, LVESD—end-systolic diameter, IVS—interventricular septum, PW—posterior wall thickness, RWT—relative wall thickness, LVM—LV mass, LVMI—LV mass index, TR—tricuspid regurgitation velocity, E—transmitral inflow velocity at early diastole, A—transmitral inflow velocity at late diastole, e’ sept/lat—septal/lateral mitral annular diastolic velocities, DT—deceleration time, LVEF—ejection fraction, GLS—global longitudinal strain, GCS—global circumferential strain, GRS—global radial strain.

**Table 3:** Comparison of Global Longitudinal Strain (GLS) and left atrial (LA) reservoir strain for different etiologies of cirrhosis in patients.

Etiology	Average GLS			LA Reservoir Strain		
Idiopathic (*n* = 7)	−22.06	±	4.16	40.91	±	15.53
Alcoholic (*n* = 25)	−20.91	±	4.12	30.28	±	10.03
HCV (*n* = 10)	−22.44	±	4.01	33.70	±	9.15
NASH (*n* = 8)	−20.05	±	4.76	33.32	±	12.82
PSC (*n* = 7)	−20.85	±	3.19	34.00	±	11.10
Autoimmune (*n* = 8)	−24.73	±	4.66	33.95	±	10.46
Others (*n* = 15)	−20.35	±	3.11	31.50	±	8.49
*p*	0.143			0.748		

HCV—Hepatitis C Virus Infection, NASH—Non-alcoholic steatohepatitis, PSC—Primarily sclerosing cholangitis

**Table 4:** Echocardiographic parameters of the left atrium.

Variables	*n*	Controls			*n*	ESLD Patients				Statistics *p*
LA Volume_max_ (mL)	30	28.85	±	17.21	80		75.13	±	49.52	<0.001
LA Volume_pre-A_ (mL)	22	16.65	±	8.64	60		40.68	±	28,90	<0.001
LA Volume_min_ (mL)	30	9.00	±	6.11	80		21.27	±	14.95	<0.001
LAVI_max_ (mL/m^2^)	30	15.37	±	8.65	79		38.73	±	24.04	<0.001
LAVI_pre-A_(mL/m^2^)	22	8.90	±	4.23	59		21.24	±	15.04	<0.001
LAVI_min_ (mL/m^2^)	30	4.83	±	3.27	79		10.90	±	7.11	<0.001
LA TotEV (mL)	30	19.85	±	13.15	80		53.86	±	38.48	<0.001
LA PassEV (mL)	22	9.96	±	6.14	60		35.71	±	32.13	<0.001
LA ActEV (mL)	22	8.45	±	5.45	60		19.81	±	18.61	0.001
LA TotEF (%)	30	68	±	17	80		71	±	11	0.872
LA PassEF (%)	22	39	±	13	60		45	±	16	0.052
LA ActEF (%)	22	51	±	18	60		48	±	16	0.213
**Strain Analysis (%)**										
Reservoir strain	30	39.97	±	9.74	80		32.86	±	10.65	0.002
Conduit strain	30	21.12	±	7.40	80		15.38	±	6.94	<0.001
Contractile strain	30	18.85	±	5.08	80		17.48	±	7.37	0.352
**Strain Rate Analysis (1/s)**										
Reservoir SR	30	1.66	±	0.44	80		1.39	±	0.40	0.003
Conduit SR	30	−1.92	±	0.72	80		−1.23	±	0.47	<0.001
Contractile SR	30	−2.43	±	0.68	80		−1.87	±	0.64	<0.001

LA—left atrium, LAVI—left atrium volume index, pre-a—pre-atrial contraction, TotEV—total emptying volume, PassEV—passive emptying volume, ActEV—active emptying volume, TotEF—total emptying fraction, PassEF—passive emptying volume, ActEF—active emptying volume, SR—strain rate.

**Table 5:** Correlation analysis in ESLD patient cohort.

Variables	*r*							
	ALAT (U/L)	ASAT (U/L)	AP (U/L)	γGT (U/L)	Creatinine (mg/dL)	Bilirubin	INR	MELD Score
**Left ventricle**								
GLS average	−0.306 **	−0.297 **	**0.065**	**0.192**	**−0.084**	−0.328 **	−0.324 **	−0.361 **
GLS mid-myocardial	−0.304 **	−0.294 **	0.071	0.190	−0.090	−0.325 **	−0.325 **	−0.360 **
GLS endocardial	−0.302 **	−0.300 **	0.080	0.198	−0.063	−0.313 **	−0.338 **	−0.346 **
GLS epicardial	−0.306 **	−0.296 **	0.037	0.173	−0.114	−0.349 **	−0.293 **	−0.379 **
**Left atrium**								
R strain	0.275 *	0.301 **	0.079	0.018	−0.271 *	0.228 *	0.162	0.111
Ct strain	0.223 *	0.316 **	0.205	0.025	−0.239 *	0.228 *	0.119	0.127
Cd strain	0.186	0.191	−0.032	−0.001	−0.155	0.160	0.107	0.058
SRs	0.249 *	0.234 *	0.004	−0.030	−0.246 *	0.198	0.179	0.144
SRe	−0.273 *	−0.217	−0.141	−0.130	0.250 *	−0.272 *	−0.014	−0.102
SRa	−0.315 **	−0.274 *	−0.024	−0.121	0.288 **	−0.105	0.055	0.068
TotEV	0.010	−0.022	−0.078	−0.174	0.296 **	0.076	0.324 **	0.273 *
PassEV	−0.040	−0.076	−0.003	−0.113	0.323 *	0.085	0.227	0.227
ActEV	−0.087	−0.147	−0.330 **	−0.158	0.155	−0.126	0.345 **	0.058
TotEF	0.282 *	0.158	0.072	−0.067	−0.145	0.102	−0.041	0.012
PassEF	0.209	0.195	0.317 *	0.070	0.090	0.281 *	−0.038	0.226
ActEF	0.317 *	0.156	−0.094	−0.088	−0.233	−0.029	0.111	−0.046

Significant correlations are given in bold for visualization purposes. * marks *p* < 0.05, ** marks *p* < 0.01. GLS – global longitudinal strain, R—reservoir, Ct—contractile, Cd—conduit, SRs—reservoir strain rate, SRe—conduit strain rate, SRa—contractile strain rate, TotEV—total emptying volume, PassEV—passive emptying volume, ActEV—active emptying volume, TotEF—total emptying fraction, PassEF—passive emptying volume, ActEF—active emptying volume, ALAT—alanine aminotransferase, ASAT—aspartate aminotransferase, AP—alkaline phosphatase, γGT—gamma-glutamyltransferase, INR—international normalized ratio (INR), MELD—model for end-stage liver diseas.

## Data Availability

The data presented in this study are available on request from the corresponding author. The data are not publicly available due to ethical restrictions.

## References

[1] Izzy M., VanWagner L.B., Lin G., Altieri M., Findlay J.Y., Oh J.K., Watt K.D., Lee S.S., Cirrhotic Cardiomyopathy Consortium (2020). Redefining Cirrhotic Cardiomyopathy for the Modern Era. Hepatology.

[2] Carvalho M.V.H., Kroll P.C., Kroll R.T.M., Carvalho V.N. (2019). Cirrhotic cardiomyopathy: The liver affects the heart. Braz. J. Med. Biol. Res..

[3] Farr M., Schulze P.C. (2014). Recent advances in the diagnosis and management of cirrhosis-associated cardiomyopathy in liver transplant candidates: Advanced echo imaging, cardiac biomarkers, and advanced heart failure therapies. Clin. Med. Insights Cardiol..

[4] Liu H., Jayakumar S., Traboulsi M., Lee S.S. (2017). Cirrhotic cardiomyopathy: Implications for liver transplantation. Liver Transpl..

[5] Dimitroglou Y., Aggeli C., Alexopoulou A., Mavrogeni S., Tousoulis D. (2019). Cardiac Imaging in Liver Transplantation Candidates: Current Knowledge and Future Perspectives. J. Clin. Med..

[6] Karki N., Kc S., Sharma D., Jaisi B., Khadka S. (2019). Cardiac Dysfunction in Patients with Liver Cirrhosis. J. Nepal Health Res. Counc..

[7] Naqvi I.H., Mahmood K., Naeem M., Vashwani A.S., Ziaullah S. (2016). The heart matters when the liver shatters! Cirrhotic cardiomyopathy: Frequency, comparison, and correlation with severity of disease. Prz. Gastroenterol..

[8] Ruiz-del-Arbol L., Serradilla R. (2015). Cirrhotic cardiomyopathy. World J. Gastroenterol..

[9] Bansal M., Kasliwal R.R. (2013). How do I do it? Speckle-tracking echocardiography. Indian Heart J..

[10] (2020). Correction. Hepatology.

[11] Gan G.C.H., Ferkh A., Boyd A., Thomas L. (2018). Left atrial function: Evaluation by strain analysis. Cardiovasc. Diagn. Ther..

[12] Pathan F., D’Elia N., Nolan M.T., Marwick T.H., Negishi K. (2017). Normal Ranges of Left Atrial Strain by Speckle-Tracking Echocardiography: A Systematic Review and Meta-Analysis. J. Am. Soc. Echocardiogr..

[13] Morris D.A., Belyavskiy E., Aravind-Kumar R., Kropf M., Frydas A., Braunauer K., Marquez E., Krisper M., Lindhorst R., Osmanoglou E. (2018). Potential Usefulness and Clinical Relevance of Adding Left Atrial Strain to Left Atrial Volume Index in the Detection of Left Ventricular Diastolic Dysfunction. JACC Cardiovasc. Imaging.

[14] Morris D.A., Gailani M., Vaz Perez A., Blaschke F., Dietz R., Haverkamp W., Ozcelik C. (2011). Left atrial systolic and diastolic dysfunction in heart failure with normal left ventricular ejection fraction. J. Am. Soc. Echocardiogr..

[15] To A.C., Flamm S.D., Marwick T.H., Klein A.L. (2011). Clinical utility of multimodality LA imaging: Assessment of size, function, and structure. JACC Cardiovasc. Imaging.

[16] Nagueh S.F., Smiseth O.A., Appleton C.P., Byrd B.F., Dokainish H., Edvardsen T., Flachskampf F.A., Gillebert T.C., Klein A.L., Lancellotti P. (2016). Recommendations for the Evaluation of Left Ventricular Diastolic Function by Echocardiography: An Update from the American Society of Echocardiography and the European Association of Cardiovascular Imaging. J. Am. Soc. Echocardiogr..

[17] Chen Y., Chan A.C., Chan S.C., Chok S.H., Sharr W., Fung J., Liu J.H., Zhen Z., Sin W.C., Lo C.M. (2016). A detailed evaluation of cardiac function in cirrhotic patients and its alteration with or without liver transplantation. J. Cardiol..

[18] Jansen C., Cox A., Schueler R., Schneider M., Lehmann J., Praktiknjo M., Pohlmann A., Chang J., Manekeller S., Nickenig G. (2018). Increased myocardial contractility identifies patients with decompensated cirrhosis requiring liver transplantation. Liver Transpl..

[19] Kim H.M., Kim H.K., Lee J.H., Lee Y.B., Park E.A., Park J.B., Lee S.P., Kim Y.J., Kim Y.J., Yoon J.H. (2020). Myocardial structural and functional changes in patients with liver cirrhosis awaiting liver transplantation: A comprehensive cardiovascular magnetic resonance and echocardiographic study. J. Cardiovasc. Magn. Reson..

[20] Mechelinck M., Hartmann B., Hamada S., Becker M., Andert A., Ulmer T.F., Neumann U.P., Wirtz T.H., Koch A., Trautwein C. (2020). Global Longitudinal Strain at Rest as an Independent Predictor of Mortality in Liver Transplant Candidates: A Retrospective Clinical Study. J. Clin. Med..

[21] Bolognesi M., Di Pascoli M., Verardo A., Gatta A. (2014). Splanchnic vasodilation and hyperdynamic circulatory syndrome in cirrhosis. World J. Gastroenterol..

[22] Zardi E.M., Abbate A., Zardi D.M., Dobrina A., Margiotta D., Van Tassell B.W., Afeltra A., Sanyal A.J. (2010). Cirrhotic cardiomyopathy. J. Am. Coll. Cardiol..

[23] Pagourelias E.D., Sotiriou P., Papadopoulos C.E., Cholongitas E., Giouleme O., Vassilikos V. (2016). Left Ventricular Myocardial Mechanics in Cirrhosis: A Speckle Tracking Echocardiographic Study. Echocardiography.

[24] Nasr F.M., Metwaly A., Khalik A.A., Darwish H. (2015). Cardiac dysfunction in liver cirrhosis: A tissue Doppler imaging study from Egypt. Electron. Physician.

[25] Cesari M., Fasolato S., Rosi S., Angeli P. (2015). Cardiac dysfunction in patients with cirrhosis: Is the systolic component its main feature?. Eur. J. Gastroenterol. Hepatol..

[26] Larsen C.M., Vanden Bussche C.L., Mankad S., Nihoyannopoulos P., Kisslo J. (2018). Principles of Measuring Chamber Size, Volume and Hemodynamic Assessment of the Heart. Echocardiography.

[27] Bansal M., Sengupta P.P., Khandheria B.K., Nihoyannopoulos P., Kisslo J. (2018). Echocardiography in Heart Failure. Echocardiography.

[28] Cameli M., Mandoli G.E., Loiacono F., Dini F.L., Henein M., Mondillo S. (2016). Left atrial strain: A new parameter for assessment of left ventricular filling pressure. Heart Fail. Rev..

[29] Vieira M.J., Teixeira R., Goncalves L., Gersh B.J. (2014). Left atrial mechanics: Echocardiographic assessment and clinical implications. J. Am. Soc. Echocardiogr..

[30] Mondillo S., Cameli M., Caputo M.L., Lisi M., Palmerini E., Padeletti M., Ballo P. (2011). Early detection of left atrial strain abnormalities by speckle-tracking in hypertensive and diabetic patients with normal left atrial size. J. Am. Soc. Echocardiogr..

[31] Morris D.A., Takeuchi M., Krisper M., Kohncke C., Bekfani T., Carstensen T., Hassfeld S., Dorenkamp M., Otani K., Takigiku K. (2015). Normal values and clinical relevance of left atrial myocardial function analysed by speckle-tracking echocardiography: Multicentre study. Eur. Heart J. Cardiovasc. Imaging.

[32] Hiebert J.B., Vacek J., Shah Z., Rahman F., Pierce J.D. (2019). Use of speckle tracking to assess heart failure with preserved ejection fraction. J. Cardiol..

[33] Sampaio F., Pimenta J., Bettencourt N., Fontes-Carvalho R., Silva A.P., Valente J., Bettencourt P., Fraga J., Gama V. (2014). Left atrial function is impaired in cirrhosis: A speckle tracking echocardiographic study. Hepatol. Int..

